# Commentary: Indoleamine 2,3-Dioxygenase-Expressing Aortic Plasmacytoid Dendritic Cells Protect against Atherosclerosis by Induction of Regulatory T Cells

**DOI:** 10.3389/fimmu.2017.00140

**Published:** 2017-02-13

**Authors:** Pasquale Maffia, Yvonne Döring, Erik A. L. Biessen, Ziad Mallat

**Affiliations:** ^1^Centre for Immunobiology, College of Medical, Veterinary and Life Sciences, Institute of Infection, Immunity and Inflammation, University of Glasgow, Glasgow, UK; ^2^BHF Centre of Excellence in Vascular Science and Medicine, College of Medical, Veterinary and Life Sciences, University of Glasgow, Glasgow, UK; ^3^Department of Pharmacy, University of Naples Federico II, Naples, Italy; ^4^Institute for Cardiovascular Prevention, Ludwig-Maximilians-University Munich, Munich, Germany; ^5^DZHK (German Centre for Cardiovascular Research), Partner Site Munich Heart Alliance, Munich, Germany; ^6^Experimental Vascular Pathology Group, Department of Pathology, CARIM, Maastricht University Medical Center, Maastricht, Netherlands; ^7^Institute of Molecular Cardiovascular Research, RWTH Klinikum Aachen, Aachen, Germany; ^8^Division of Cardiovascular Medicine, Department of Medicine, University of Cambridge, Addenbrooke’s Hospital, Cambridge, UK; ^9^Unit 970, Paris Cardiovascular Research Center, Institut National de la Santé et de la Recherche Médicale, Paris, France

**Keywords:** atherosclerosis, plasmacytoid dendritic cells, aorta, antigen presentation, regulatory T cells, type I interferon, indoleamine 2,3-dioxygenase

Plasmacytoid dendritic cells (pDCs) are a unique subset of dendritic cells gaining increasing interest in atherosclerosis because of their ability to influence disease progression through a broad range of activities. Positioned at the border between innate and adaptive immunity, pDCs are found in lymphoid and somatic tissues, where they play different roles depending on anatomical location and context (1).

Several lines of evidence in recent years have highlighted the contribution of pDCs to the development of atherosclerosis. Circulating pDCs are reduced in patients with coronary and peripheral artery diseases (1, 2); on the contrary, although scarce, pDCs become detectable in human atherosclerotic lesions, where their levels are either considered to be stable [based on blood dendritic cell antigen 2 (BDCA2) staining] or tend to increase (based on CD123 staining) with disease progression (1, 3–5). A recent study also suggested a causal association between pDC-derived type I IFN and the development of atherosclerosis in systemic lupus erythematosus (6).

Results from experimental animal studies were more conflicting; however, most of the data point toward a detrimental role played by pDCs in atherosclerosis. Initially, using an antibody-mediated depletion model, pDCs were shown to protect against atherosclerosis and vascular response to injury induced by bilateral placement of semiconstrictive collars in the carotid arteries of low-density lipoprotein receptor (Ldlr)^−/−^ mice (3). However, subsequent studies demonstrated that antibody-mediated depletion of pDCs inhibited experimental atherosclerosis in apolipoprotein-E (apoE)^−/−^ mice (4, 7). More recently, by using a CD11c-restricted deletion of the transcription factor Tcf4/E2-2, which is required for pDC development and maintenance, pDC deficiency was shown to reduce plaque formation in Ldlr^−/−^ chimeras (8). This phenotype was confirmed in chimeras lacking the pIII and IV promoters of MHC-II transactivator (CIITA) in pDCs, leading to MHC-II-restricted antigen presentation defects in pDCs. These data support a proatherogenic role for pDCs in experimental atherosclerosis and identify a critical role played by pDCs in sustaining atherogenic T cell response(s).

The proatherogenic role of pDCs has been challenged by the recent article published in Cell Metabolism (9), where the authors suggested that pDCs may exert a tolerogenic role in experimental atherosclerosis, being able to decrease plaque progression *via* induction of regulatory T cells (Tregs).

The authors performed a difficult and elegant work in the identification of a defined pure population of pDCs in the mouse aorta, being able to demonstrate for the first time, the capacity of aortic murine and human pDCs to produce type I IFN in both healthy and atherosclerotic vessels. The authors confirmed the scarce presence and anatomical location of pDCs in the intimal space and adventitia, as previously demonstrated (3, 7, 8); moreover, total aortic pDC numbers increased with atherosclerosis progression.

Importantly, the authors demonstrated that treatment with pDC-targeting Ab [plasmacytoid dendritic cell antigen-1 (BST2/PDCA-1/CD317)], previously used to investigate pDCs in experimental atherosclerosis, was not fully selective, leading to partial (albeit not significant) aortic macrophage depletion. Previous immunofluorescence staining of aged apoE^−/−^ mouse atherosclerotic arteries in our hands showed that a significant number of PDCA-1^+^ cells were present in the T cell area of arterial tertiary lymphoid organs, with localization of PDCA-1 also observed in endothelial cells of high endothelial venules (10). Yun et al. (9) work complements our studies, directly demonstrating that antibody-mediated pDC depletion should be avoided in atherosclerosis research, given that PDCA-1 is promiscuously expressed in the vascular inflammatory environment.

The key experiments performed by the authors to assess pDC contribution to experimental atherosclerosis present incongruences that merit discussion.

## Efficacy of pDC Depletion after Prolonged Treatment in BDCA2-Diphtheria Toxin Receptor (DTR) Mice

To explore the role of pDCs in plaque formation, they generated atherosclerotic chimeric mice by reconstituting Ldlr^−/−^ mice with bone marrow from BDCA2-DTR mice. These transgenic mice express a simian DTR under the transcriptional control of the human pDC gene promoter BDCA2 (11). In these animals, pDCs (SiglecH^+^B220^+^) are successfully and selectively depleted 24 h after diphtheria toxin (DT) treatment [administered intraperitoneally (i.p.) at 100–120 ng/mouse] (11). In the original research article, DT-induced depletion persisted for 2–3 days (11). It was also suggested that pDCs could be depleted for longer periods with repeated DT administration, but no data were shown to support this statement. Several other studies have used BDCA2-DTR mice; nevertheless, only one of those studies depleted pDCs for more than 2–3 weeks. In particular, pDCs were said to be depleted in BDCA2-DTR mice for 8 weeks in a lupus model; however, only splenic depletion data at 24 and 72 h were shown (12).

More recently, BDCA2-DTR mice have been crossed with apoE^−/−^ mice to generate apoE^−/−^ BDCA2-DTR mice for the investigation of pDCs in atherosclerosis (13). DT administration to induce pDC depletion for 4 weeks (0.01 mg/kg administered i.p. three times per week) during high-fat diet (HFD) did not affect lesion formation in the aortic sinus. Importantly, detailed analysis of depletion efficiency demonstrated complete pDC depletion for 1 week only. After 4 weeks of continuous DT administration, pDCs failed to express DTR anymore and, therefore, could not be depleted. On the contrary, the authors showed an increase in B cells in all organs, accompanied by changes in the inflammatory profile in pDC-“depleted” mice compared to controls. Although we cannot exclude that the breeding of BDCA2-DTR mice to apoE^−/−^ may have contributed to altered pDC depletion, the available data highlight the importance to carefully evaluate depletion efficiency in long-term experiments with BDCA2-DTR mice.

Yun et al. (9) used chimeric Ldlr^−/−^:BDCA2-DTR mice fed with HFD for 12 weeks, with or without DT treatment (0.5 μg/mouse per week, i.p.), to deplete pDCs. Atherosclerotic plaque formation significantly increased in the sinus of pDC-depleted animals. Depletion efficiency was shown in BDCA2-DTR mice administered with DT twice at 24 h intervals. Similarly, selective depletion was shown in Ldlr^−/−^:BDCA2-DTR mice; however, the time point of assessment of aortic and spleen pDC depletion was not reported in the original manuscript. Subsequently, data showing partial pDC depletion in the spleen, after 12 weeks of DT injection, have been added as corrigendum to the original paper. However, pDCs were depleted in only 60% of the mice, and how long the depletion lasted after each DT injection was not shown. This is of particular relevance given that DT was only administered once per week. In addition, depletion efficacy in the aorta was not assessed at this later time point. As discussed above, it would have been fundamental to evaluate the efficacy and kinetics of pDC depletion in all target organs throughout the duration of the experiment. This represents a limitation of the study given that previous studies were unable to provide conclusive evidence of effective and continuous long-term pDC depletion in BDCA2-DTR mice.

Similarly, it would have been important to assess selectivity of the depletion in atherosclerotic mice following 12 weeks of HFD. pDCs are acutely and selectively depleted in BDCA2-DTR mice in both aorta and spleen in the steady state. However, on the one hand, TLR7/9 agonists have been shown to downregulate expression of BDCA2 in pDCs (14), suggesting that activated pDCs in advanced atherosclerotic vessels may be less susceptible to depletion; on the other hand, the truncated human BDCA2 promoter could give rise to off-target DTR expression, particularly in the advanced stages of atherosclerosis, where promiscuous expression of BDCA2 by other leukocyte subsets may occur.

## Toxic and Off-Target Effects of DT Treatment

Importantly, controls for the DT treatment were not entirely adequate. Eight weeks following chimera generation, Ldlr^−/−^:BDCA2-DTR mice were fed with HFD and injected with PBS or DT for the subsequent 12 weeks. Treatments with mutant DT or DT-treated Ldlr^−/−^:WT-DTR^-^ chimeric mice were not used as controls to assess any potential toxicity related to DT treatments, as conventionally performed (15, 16). Mice are generally resistant to DT; however, several reports highlight potential DT toxic effects (17) and development of anti-DT antibodies (18). In addition, off-target effects have been observed following DT injection. For example, CD11c-DTR and CD11c-DOG mice developed neutrophilia 24 h after DT administration, which is independent from DC depletion (19). Moreover, ectopic DTR expression in DTR Tg mouse models may also lead to toxic effects of DT. For example, DT has been shown to be lethal in Zbtb46-DTR mice, due to aberrant expression of Zbtb46 in erythroid progenitors and endothelial cells (20).

In a similar experimental setting to Yun et al. (9), Biessen and coworkers observed no significant changes in lesion formation in Ldlr^−/−^:BDCA2-DTR mice fed with HFD and injected with DT or mutant DT (120 ng/mouse three times per week, i.p.) for 3 weeks (unpublished data). The experiment was terminated due to significant decrease in body weight and granulocytosis observed in blood and spleen of DT-depleted mice at end point. These data confirm the importance to adequately control DT administration in experiments with DTR Tg mice.

## Merits and Pitfalls of the Mechanistic Studies

Finally, the authors found that mouse and human aortic pDCs expressed the tolerogenic enzyme indoleamine 2,3-dioxygenase (IDO)-1, suggesting an activated/matured pDC phenotype. Furthermore, they propose a direct functional link between the presence of IDO-1^+^ pDCs and Treg generation in the diseased vessel. These data align with several earlier findings, including in the context of atherosclerosis, Daissormont et al. (3). However, both the latter study and that of Yun et al. (9) provided no demonstration of a direct effect of pDCs or IDO expression by pDCs on vascular Tregs *in vitro* or *in vivo*. First, the authors showed an interesting correlation between aortic pDCs and Treg numbers with disease progression. Second, they demonstrated reduced Treg numbers in Ido1^−/−^ mice compared to WT. However, in Ido1^−/−^ mice, the knockout effect is not pDC restricted/specific and, therefore, we cannot rule out potential contributions from other leukocyte subsets known to express IDO, such as conventional DCs, macrophages, and smooth muscle cells. Finally, the authors demonstrated that depletion of pDCs in atherosclerotic mice resulted in reduction of aortic Tregs. These findings are interesting; however, implications are difficult to be interpreted given that no data on other T cell subsets were shown. Similarly, the authors demonstrated that antigen presentation by pDCs induced OVA-specific Tregs in the aorta of Ldlr^−/−^:OT-II chimeras. MHC class II-restricted antigen presentation by pDCs may lead to the expansion of all CD4^+^ Tg T cell subsets (that have not been investigated), and the effect may not, therefore, be restricted to Tregs.

## Lessons Learned and Proposed Next Steps

The abovementioned limitations highlight the importance to deplete specifically and continuously pDCs *in vivo*. All the genetically modified mouse models developed to date present limitations, and therefore, new selective, reproducible, and advanced tools are urgently required. pDCs may act in an immunogenic or tolerogenic manner in atherosclerosis depending on the specific anatomical niche, the local inflammatory environment, and the different stages of the pathology. Therefore, understanding the delicate balance between pDC-driven immunogenic and tolerogenic response(s) is becoming increasingly crucial. At the same time, it would be key to understand the net contribution of vascular vs. systemic pDCs to disease development and progression and investigate why several leukocytes acquire a “pDC-like” phenotype in atherosclerotic arteries. As these studies progress, we will learn more about whether pDCs are indeed viable diagnostic, prognostic, and/or therapeutic targets in atherosclerosis.

## Author Contributions

PM and ZM drafted this commentary. All authors listed have made substantial, direct, and intellectual contribution to the work and approved it for publication.

## Conflict of Interest Statement

The authors declare that the research was conducted in the absence of any commercial or financial relationships that could be construed as a potential conflict of interest.
